# Hearts and minds: The technopolitical role of affect in sociotechnical imaginaries

**DOI:** 10.1177/03063127241257489

**Published:** 2024-06-06

**Authors:** Stephen Hughes

**Affiliations:** University College London, London, UK

**Keywords:** sociotechnical imaginaries, affect, emotion, psychoanalysis, fracking

## Abstract

Sociotechnical imaginaries (SIs) have emerged as a popular and generative concept within Science and Technology Studies (STS). This article draws out the affective component of SIs, combining a review of relevant literatures with an empirical case study of an anti-fracking imaginary in Ireland to suggest how we might theorize an affective technopolitics of SIs. The literature review identifies three key aspects of SIs that would benefit from a more coherent conceptualization of affect: the utopian, productive, and collectivizing dimensions of imaginaries. Emotions such as desire and fear appear prominently in the SI literature, but in ways that require development. Using empirical examples from my research, I outline what this developed understanding of emotions in imaginaries might look like. I examine the role that emotions played in the development and settlement of an anti-fracking imaginary in Ireland, highlighting how the intensive, multimodal, and dynamic nature of affect underpinned the productive, collective, and utopian dimensions of the SI. I conclude with some remarks about how this developed theory of emotion positions STS researchers to address issues of humanity, representation, and the building of better worlds.

We did it by engaging the community, through participation and empowerment. We are proud of where we are from. We are proud of Leitrim and Ireland. We wanted to reflect what Leitrim was about, farmers, fishermen, artists, professionals, parents and about sustainability. This is about Ireland. We knew we wouldn’t win unless we brought everyone along. We understood that we had to convince everyone. We knew that we had to be non-political. We had to win over hearts and minds.

— Anti-fracking campaigner, Leitrim Observer (2017)

In 2017, a piece of legislation passed both houses of the Oireachtas (Irish legislature), prohibiting fracking in the Republic of Ireland,^1^ marking a significant point in the realization of an anti-fracking sociotechnical imaginary (SI) in Ireland. This was an imaginary that had developed counter to the dominant vision for Ireland’s energy future, in which oil and gas companies and the Irish Government expected fracking to play an important role. During a debate in Dáil Éireann (the lower house and principal chamber of the Oireachtas) Deputy Eugene Murphy, of the Fianna Fáil party, stated that ‘the people who have us here [are] the people from Leitrim and north Roscommon who started this campaign … the real reason we are here is that those people brought it to our attention and have fought very hard to get this done’ (Murphy, 2017). Between 2014 and 2017, I conducted research with these people—farmers, builders, teachers, parents, and owners of small local businesses—who had gathered together to oppose the introduction of fracking to Ireland. The startling success of the counter-imaginary, I suggest, was as much to do with its affective dimension as its epistemic and material dimensions.

What stands out in this case study is the intensity of feeling about fracking and how it engineered change, the manner in which local practices of embodied and multimodal meaning-making drew people together, and how painful conflicts within the utopian vision of the future were managed within the campaign. The current understanding of sociotechnical imaginaries in Science and Technology Studies (STS) struggles to explain these aspects of the anti-fracking imaginary in Ireland, as it does not have an adequate theory of the role that affect plays in the productive, collective, and utopian dimensions of shared technosocial visions. While affect is frequently referenced in SIs literature and case studies, it is relatively undertheorized and lacks a nuanced account of the distinctive technopolitical characteristics of emotions.

This paper aims to address this gap and illuminate how the anti-fracking case study can provide us with grounds for thinking about the role emotions play in collective imagination, in powering change, drawing people together, and managing conflicting hopes and fears.

## How is affect theorized in sociotechnical imaginaries scholarship?

### What is a sociotechnical imaginary?

A sociotechnical imaginary is an analytic device deployed by social scientists to capture the various ways that social groups collectively imagine their relationship with science and technology and the futures these relationships might bring about. It is also the co-produced object of inquiry in these studies—the assemblage of discourses, representations, institutions, and social practices that constitutes such a collective vision. The standard definition of a sociotechnical imaginary is Jasanoff’s (2015, p. 4): ‘collectively held, institutionally stabilized, and publicly performed visions of desirable futures, animated by shared understandings of forms of social life and social order attainable through, and supportive of, advances in science and technology’. The concept has been deployed in STS to answer questions about the political and constitutive relationships between technoscience and individuals, groups, and their various arrangements and practices. These are questions about:

- The utopian function of imaginaries: shared commitments to ‘how life ought to be lived and what constitutes the “good life”’ (Tutton, 2021, p. 419).
- The productive capacity of imaginaries: ‘how sociotechnical projects travel from imagination and conception to realization’ (Sovacool & Hess, 2017, p. 719).
- The collectivizing nature of imaginaries: the roles played by science and technology ‘in connecting the individual’s subjective self-understanding to a shared social or moral order’ (Jasanoff, 2015, p. 5).

Imaginaries hold normative power in their capacity to challenge the present while offering alternative visions of the future. In this way, we can think of SIs as having a utopian function. A utopian vision is ‘a specific form of creative and transformative reflection that breaks the spell of the status quo, a demonstration—in thought and in practice—that things could be otherwise’ (Thaler, 2022, p. 3). As Tutton (2021) points out, the utopian dimension of imaginaries seeks to ‘challenge deterministic narratives of progress, to show how there is not one but multiple imaginaries and how each are open to resistance and contestation’ (p. 419). Utopian thinking isn’t *a priori* virtuous, it involves a politics of its own. As Sovacool and Hess (2017) put it, ‘one person’s utopia can be another’s dystopia’ (p. 720). Thaler (2022) acknowledges this in his account of utopianism, arguing that ‘all utopian visions need to be scrutinized against the material and ideological background in which they are formulated’ (p. 29). This involves critique of tendencies towards totalitarianism and magical thinking, with the former describing an imaginary’s attempt to universalize a singular vision of the good life, and the latter referring to imagination as wishful fantasy with no concrete steps available for implementation. To guard against these two modes of utopianism, Thaler suggests that utopian thinking involve the ‘education of a desire for being and living otherwise’ (p. 3). I take this education of desire to be a form of emotional learning, to which I return later. For now, I turn to the productive dimension of SIs.

SIs do not ‘merely’ imagine alternative ways of living and being, but play active roles in materializing those alternatives. In projecting an image of ‘the kind of society that sociotechnical innovation can bring into being and the kind of society that is needed for innovation to happen’ (Tutton, 2021, p. 418), SIs steer collectives down certain technoscientific pathways (Levidow & Papaioannou, 2013, p. 38). They provide an answer to the question posed by Jasanoff (2015) as to ‘why, at significant forks in the road, societies opt for particular directions of choice and change over others and why those choices gain stability or, at times, fail to do so’ (p. 14). Imagination serves a pivotal role in the organization and structure of critical thought. It is through imagining that individuals and groups are able to think about how to arrange technosocial life differently. McNeil et al. (2017) suggest that the affective dimension of imaginaries provides them with this productive political capacity, surpassing more restricted concepts like interests or ideologies, which ‘operate primarily in a cognitive register’ (p. 457). However, this affective dimension remains underexplored.

Imaginaries hold enormous power in drawing collectives together and shaping social order. The theory of imaginaries is rooted in thinking about how societies imagine themselves. Said’s (1977) pioneering work on ‘imaginative geography’ claimed that global spatial scales (East and West) could be delineated and ordered through collective visions of self and other. Sociotechnical imaginaries have brought broader political insights about social imaginaries into conversation with STS by focusing on the role that science and technology play in the sociomaterial enactment of modernity (Jasanoff, 2015).

I do not seek to refute the core ideas of the sociotechnical imaginary. Rather, I would like to extend them to take better account of emotion and multimodal forms of meaning-making to understand the normative, productive, and social dimensions of collective imagination. Before making that argument, I would like to provide a brief overview of the ways that emotions are currently theorized in work on SIs, followed by two theoretical approaches to affect that offer greater scope for viewing the normative, productive, and collective dimensions of sociotechnical imagination.

### How are emotions currently theorized?

This section briefly outlines the limited way that affect is theorized in the SIs literature, and how we can begin to think about the technopolitical function that emotions serve in shared visions of the future. We will see that despite the prominence of affects such as desire, hope, anxiety, and fear in SIs literature, there is scope for a deeper account of their role and function in powering imaginaries. I show that emotions are typically restricted to discourse, obscuring the embodied and multimodal dimensions of shared practices of meaning-making and how this meaning-making draws people together. Finally, we will see how emotions are typically presented in restricted relational terms as binary pairs (such as desire and fear) which draw collectives either towards or away from certain futures. I discuss sporadic research that is beginning to display the work that affect is performing in Sis, but which thus far lacks systematic theorization. Finally, I explore the distinctively technopolitical character of emotion in SIs.

Hurlbut (2015a) describes how the legacy of Asilomar marks a powerful SI which has been successful at ‘managing public alarm’ (p. 127). He points to a 2002 *Science* commentary that states how, as a result of the conference, ‘hope prevailed over fear’ (Hurlbut, 2015a, p. 127). Hurlbut (2015a) traces this sentiment in a broader imaginary of scientific responsibility which he terms ‘governable emergence’ (p. 127). At stake is a balance of emotional registers, whereby science can ‘declare what technological futures are possible, desirable, and good’ (Hurlbut, 2015b, p. 113). The dynamics of these emotive registers—hope, desire, fear—are not interrogated; they are left largely intact as black boxes. Instead, Hurlbut examines the various rational arguments underpinning the sociotechnical imaginary of governable emergence.

Elsewhere, the emotional dimensions of collective imagination are largely reduced to discourse. Jasanoff and Kim (2009) suggest that language is ‘a crucially important medium for the construction of imaginaries’, prompting them to examine the ‘discursive elements’ of policy narratives (p. 122). This discursive approach to tracing SIs and the emotions associated with them is followed by many others (e.g., Ballo, 2015; Levenda et al., 2019; Schiølin, 2020; Tozer & Klenk, 2018), who feature studies of policies, regulations, public hearings, planning documents, white papers, and governance texts. What are often missing are empirical accounts of emotions and feelings. This scholarship on discourses is important and worthwhile. However, I believe that there is an opportunity to conduct a deeper analysis to better understand how the moral orders of e.g., governable emergence, attach to individuals and collectives through affective practices.

Hope, desire, anxiety, and fear are common affects discussed in the literature. Building on work on energy imaginaries, we find desires drawing collectives towards behavioural change (Ballo, 2015), action on national security (Levenda et al., 2019), the fourth industrial revolution (Schiølin, 2020, p. 542), carbon-neutral futures (Tozer & Klenk, 2018) socioeconomic transition ‘to a “green” future’ (Kuchler, 2014, p. 431) and renewable energy projects in the Global South (Cloke et al., 2017). Hopes and fears are frequently opposed: Sartori and Bocca (2022) outline the binary of ‘hope and fears associated with AI and robots’ (p. 444); Tesfamichael (2022) examines how energy imaginaries linked to the Grand Ethiopian Renaissance Dam create polarizing tensions between hope and distrust; Dennis (2015) describes the fearful ‘monsters’ inhabiting historical US science policy, such as Lysenkoism, which ‘threaten the performance and reaffirmation of desired social orders’ (p. 56); Au (2020) describes how Chinese imaginaries of precision medicine produce ‘anxieties linked to China’s demographic and developmental transition’; Kim (2014) describes South Korean fears around stem cell research and its potential to produce an undemocratic and unjust future; and Graf and Sonnberger (2020) outline how publics are imagined as holding ‘irrational fears’ about the autonomous vehicles.

In the above examples, the emotional dimension of collective imagining is represented through the polar valences of desire and fear. Each of these valences drives communities towards (desire) or away (fear) from a given sociotechnical future or assemblage. Tutton (2021) has outlined how a binary conception of imaginaries can draw out attention away from the dynamic relationships between what can appear to be quite separate emotions. Using Berlant’s (2011) theory of cruel optimism, Tutton shows that imaginaries are contradictory and ambivalent, in that they can move us towards desired objects that block us from flourishing. Other scholars draw from resources in studies of affect to examine the role of feeling, embodiment, and multimodality in SIs. However, these accounts are scattered and fragmented, lacking a synthesis of what affect might bring to the imaginary. Individually, they offer interesting cases for illustrating the potential value of accounting for affect in SIs: Quinlan (n.d.) reveals a complex history of techno-optimism surrounding rape-kit technology in the US; Hudson (2020) explores the ambivalent imaginaries available to people seeking assisted reproduction through donated eggs; Shaw and Mykitiuk (2023) explore the multimodal dimension of the sociotechnical imaginaries they believe may be introduced by three-dimensional bioprinting; and Karpin and Mykitiuk (2021) examine how disability is figured in the imaginaries that are given shape by the reproductive projects and parental desires facilitated by the bio-medical techniques and practices of assisted reproductive technology.

What distinguishes SIs from broader uses of the term ‘imaginary’ is the unique relationship between collective imagination, science, and technology (Jasanoff, 2015). Hecht (2011, p. 3) describes technopolitics as ‘hybrid forms of power embedded in technological artifacts, systems, and practices’. Affect is a key feature of the ‘imagined preferred ways of living, value structures, and social order’ that are co-constituted with technoscientific projects (Felt, 2015, p. 2014). The next section will present two perspectives on affect—affective practice and psychosocial studies— that can help us theorise the role that emotions play in these complex technosocial arrangements.^2^

### What is emotion? What is affect?

Studies of emotion and affect are often prefaced with the claim that there is little agreement on what exactly these words mean (Scherer, 2005). This is not just an issue with the humanities and the social sciences; As Plamper (2017) points out, in ‘English-language experimental psychology, ninety-two different definitions of emotion have been counted between 1872 and 1980’ (p. 11). While there is relative consensus within the affective sciences about some of the characteristic ways in which emotions are patterned within the body (Shuman & Scherer, 2015), this is problematized by a much more confusing account of the relationship between psychobiology and ‘the dynamic figurations and assemblages of the person in social interaction, in social formations and throughout the history of individual life’ (Wetherell, 2012, p. 50).

Affective practice can be defined simply as ‘embodied meaning-making’ (Wetherell, 2012, p. 4), or in more complex terms, as ‘a figuration where body possibilities and routines become recruited or entangled together with meaning-making and other social figurations’ (Wetherell, 2012, p. 19). This second definition needs unpacking. Wetherell considers the body as a kind of figuration, a material semiotic site where its biological possibilities (and limitations), and the relational social practices that it routinely performs, come together with culturally shared meanings to co-produce emotions. Crucially, emotions are multimodal and do not begin and end in the body. They are situated and experienced at various levels, scales, and complexity—from the intense and somewhat short-lived subjective feeling of a panic attack to the inchoate, collective and enduring experience of national trauma. In this context, emotions are assembled through interweaving multimodal patterns, gaining and losing strength through repetition and connection. In these patterns, ‘somatic, neural, phenomenological, discursive, relational, cultural, economic, developmental, and historical patterns interrupt, cancel, contradict, modulate, build and interweave with each other’ (Wetherell, 2012, p. 14).

Wetherell eschews a strict conceptual or methodological division between emotion and affect. As in the affective sciences, affect is understood as a broad umbrella term referring to the various ways that ‘human emotion’ appears in social life (Wetherell, 2012, p. 4). I will be following this convention. I will refer to affect when talking broadly about the various ways that human emotions appear in the data or are spoken about in the literature. I will speak of emotions when referring to more specific instances of embodied and multimodal meaning-making. Affective practice thus includes the various ways that emotions are *done* and what they *do—*how they ‘build psychologies, identities, reputations and subjectivities as they make meaning, just as they build social orders, histories and institutions’ (Wetherell, 2012, p. 90). It is in this sense that affective practice can be integrated with sociotechnical imaginaries, as both seek to examine ‘how affective-discursive practices spatialize, demarcate and place communities and social groups’ (Wetherell et al., 2015, p. 60). However, I depart with Wetherell on her account of subjectivity, which I feel does not offer enough detail to be used effectively. Psychoanalytic psychosocial studies offers a much richer framework for understanding the relationships between individuals and collectives in shared social life.

Hoggett (2019, p. 9), has observed that ‘our collective equanimity in the face of the unprecedented risk posed by climate change is perhaps the greatest mystery of our age’. How, he asks, can most people remain calm and composed as we travel towards the one future that so many agree we do not desire? The answer, for psychoanalytic psychosocial researchers, lies in the fact that our desires and fears are in conflict, and that we employ unconscious management strategies to defend against the discomfort that these conflicts cause. These defences minimize our anxiety by blocking out aspects of reality. Weintrobe (2013, p. 9) sums up the emotional conflicts underpinning climate change: ‘In the West we feel narcissistically entitled to consume what we want from wherever we want when we want, and we also simultaneously want to protect the environment. Facing this conflict and working it through would involve facing our destructiveness towards the environment and towards our own minds.’ Rather than confront this uncomfortable reality, individuals and groups employ defensive strategies such as splitting and idealization to manage the guilt, uncertainty, and anxiety that climate change creates. Splitting involves fantasies of ‘black-and-white, all-or-nothing’ thinking where the world is split between loved and hated aspects (Weintrobe, 2013, p. 11). Idealization involves fantasies that project an all-good image onto the self or another person or group. Hinshelwood (2013) describes how the divisiveness of idealization, and its corollary, demonization, creates ‘the familiar us-and-them pattern’ (p. 166). While these defences are successful at reducing our anxiety, they can result in a distorted view of reality, allowing those in the West, for example, to continue supporting or accepting a form of life that is ultimately destructive.

Psychosocial researchers draw on psychoanalytic theory to propose ways of responding to the overwhelming anxieties that the contradictions of climate change bring about, suggesting that people work through their emotions in a psychotherapeutic manner.^3^ This involves confronting difficult and conflicting emotions in a safe and encouraging space to achieve a proportionate sense of reality. This would be achieved by avoiding magical thinking, recognizing limits, acknowledging guilt and anxiety, and creating space for gratitude, love, and repair (Hoggett, 2019; Weintrobe, 2013). It requires emotional growth as well as epistemic growth. Right now, climate change discourse is primarily geared towards producing epistemic growth—knowledge about climate change—with little attention paid to the kind of emotional growth required to make that knowledge meaningful (Jasanoff, 2010).

Psychoanalysis uses the term, ‘dynamic’ to refer to the back-and-forth relational movement of emotions as they are experienced and managed. A defining feature of this dynamic process is the idea that, for much of the time, individuals and groups are unaware that they are managing their feelings in this way. Instead, the underlying emotions and management strategies remain unconscious. That feelings are unconscious means broadly that ‘the psyche cannot be reduced to the conscious domain and that certain “contents” only become accessible to consciousness once resistances have been overcome’ (Laplanche & Pontalis, 1973, p. 475). It is because ‘unconscious ideas are pushing for expression and being kept out of awareness’ (Frosh, 2012, p. 45) that psychoanalysts refer to them as being dynamic. Crucially, for psychosocial researchers, unconscious dynamics do not simply play out in individuals’ heads. They are inherently relational, a consequence of attempts to ‘resolve the contradictions of social experience intersecting with what seem to be personal desires’ (Frosh, 2019, p. 105). Psychosocial studies attempts to strike a balance between psychological and social explanations of emotion, whereby ‘the agentic subject can be more than just an epiphenomenon or “fold” of the social’ (p. 104).

Wetherell’s affective practice offers a broad view of the various domains of affect as they appear across social life. Where the concept is weakest—the subjective—the psychoanalytic psychosocial perspective is helpful. For the purposes of this paper, we can think of affective practice as an umbrella term that can account for the variety and scale of emotion, with psychosocial theory offering more nuanced details of the dynamics at play within and between individuals. As we will see below, the power and scale of fracking resonates affectively, spurring a counter-imaginary. Resistant orders of value are enacted through practices of grief and love that use the same landscapes that fracking threatens, and the management of desire and anxiety produces a unique dynamic between tradition, modernity, utopia, and dystopia. The distinctive technopolitical characteristics of emotions, I argue, reside in their intensity, multimodality, and dynamics.

## Environmental controversy in Ireland

My research explores how fracking was collectively imagined and politically navigated in Ireland. Early on, I noticed that SIs as an analytic concept struggled to account for the ambivalence, intensity, and richness with which fracking and its futures were envisioned. When I began to integrate methodological and analytic insights from studies of affect, a more nuanced, colourful, and dynamic SI began to emerge.

The *Petroleum and Other Minerals Development (Prohibition of Onshore Hydraulic Fracturing) Act* 2017, was the result of a hard-fought campaign involving rural communities from the Northwest of Ireland. The possibility of fracking seemed to appear quite suddenly in 2011 with the awarding of onshore petroleum licensing options to three companies who sought to evaluate the potential of shale gas in the area. Encouraged by environmental campaigners, local groups formed in opposition to what they felt was a concerted effort by a host of powerful actors (Irish government, Environmental Protection Agency, oil and gas companies, international engineering consultants) to implement fracking with no democratic accountability.

This was not the first environmental controversy in Ireland and needs to be understood as part of an ongoing struggle over the shape of Ireland’s energy future, formed in part through the tensions that have arisen between the country’s values as a postcolonial state and its more recent emergence as a liberal economy. As a postcolonial nation, Ireland cherishes its independence, and has developed powerful romantic myths about the West of Ireland as a land untouched by modernity (Brereton, 2006; Gibbons, 1996). Alongside Ireland’s emergence as a liberal economy came European money and major infrastructural projects, such as roads, incinerators, and gas pipelines (Leonard, 2008). It resulted in the arrival of overseas workers who provided labour to build the burgeoning economy, and interest from foreign companies in Ireland’s English-speaking workers, natural resources, and low corporate tax rate). At times, these tensions intensified, becoming large-scale conflicts and increasingly framed as environmental controversies.

It is within this context that the campaign against fracking emerged. Communities in the Northwest of Ireland, particularly in border counties such as Leitrim and Fermanagh, were concerned about the impacts of fracking on the environment and human health. Various campaign groups were formed in the early 2010s, the most notable being *Love Leitrim*. This group was composed of locals who saw themselves as ordinary people taking on the Irish government, the Environmental Protection Agency, and engineering and investment companies with an interest in unconventional oil and gas exploration. *Love Leitrim*’s members had diverse political views, but saw themselves as united against fracking. The central aim of the group was to ‘ban fracking on the island of Ireland’ (Love Leitrim, 2017).

## Methods

The anti-fracking imaginary that I present is a case study for considering the role of emotion in collective imagining. I have used a range of methods to capture different registers of affect, including unstructured interviews, participant observation, and visual media analysis. I paid attention to ‘domains’ of affect, those instances where ‘the body has been more intrusive than it ordinarily is’, or where ‘there is notable talk occurring about emotion and feelings’ (Wetherell, 2012, pp. 96–97). I am seeking to interpret SIs as encompassing a patterned coalescence of affective-discursive work. This involves a slight shift in the methods typically used to detect imaginaries. Rather than focus solely on discursive texts and the sociomaterial mediators through which they circulate, I also explore affect across a variety of situations, memories, and images. Discourse is still considered a key mediator of affect, but alongside more body- and object-centred practices observed at events, gatherings, and drives through the countryside.

I use unstructured interviews because they offer a flexible approach to engaging participants about a topic, allow participants to direct the conversation towards issues of relevance and meaning to them, and mitigate my own bias towards discussing emotion in relation to fracking (Simons, 2014). I conducted unstructured interviews with ten *Love Leitrim* campaigners (four women and six men), interviewing five participants more than once in the interest of getting a broader sense of their experience (Cartwright, 2004) until I reached theoretical saturation (Guest et al., 2006). Each participant signed a consent form and plain language statement according to university research ethics committee requirements. My sampling method was largely iterative, in that participants in later interviews were identified by contacts made during earlier interviews (Goodyear-Smith et al., 2003). This was a necessary limitation of studying such a closed group of participants. Participant observation was guided by a desire to capture affective practices at different scales of activity, such as public talks, drives with participants, artistic exhibitions, campaign meetings, press conferences, artistic performances, and other public gatherings. To broaden the range of affective domains, I examined a selection of *Love Leitrim*’s visual media. The aim was to examine how the anti-fracking imaginary was extended, affectively, through publicly circulated images and videos.

## Affect in the anti-fracking imaginary: Intensity, multimodality, dynamics

### Intensity

Campaigners describe feeling shock at the arrival of fracking to the Northwest of Ireland. One research participant told me that they ‘couldn’t believe that anything like this could come to an area like Leitrim. There were a lot of people and they were kind of shell-shocked.’ This feeling of shock was frequently remarked upon, describing a suddenness and intensity with which fracking appeared. ‘All of a sudden, a gas company turned up in Carrick-on-Shannon and said, “we’re going to be drilling here next year and we’re going to be rolling out a huge production of oil and gas”’, one campaigner said. Another explained how, ‘all of a sudden this is the place that they want to come and start fracking on this hill behind me!’

The feeling of shock at the arrival of fracking was an important moment in the anti-fracking imaginary. In terms of temporality, it created an affective mark between past and future, configuring an Edenic ‘before’ that was shattered by an apocalyptic ‘after’. Shock becomes part of the ‘interpretive repertoire’ of emotions; a shared ‘kind of backdrop, available to be called in to formulate and describe any instance of affect’ (Wetherell, 2012, p. 94). Shock becomes an agreed way of feeling about fracking that does some work in the creation of a shared timeline of events.

This is echoed in a dance performance and accompanying poem written in solidarity with the fracking campaign (Tahany Academy, 2012). *My Farm I Adore* (Love Leitrim, 2014) was created by the local Tahany Dance Academy and plays out the story of the arrival of fracking in Leitrim and the community’s response. The performers, all children, play a variety of roles: animals (sheep, chickens), local farmers bearing ‘Farming Not Fracking’ signs, and the fracking industry holding signs with Euro currency symbols on them. A poem introducing the performance tells the story of the dance:

The cockerel crows—a sign of a new day,

the farm animals wake and begin to play.

The local farmer tends to his land,

When the postman comes by and shakes his hand.

Gives him his post and as he’s on his way,

the farmer reads it, he begins to sway.

The poem represents the ‘moment’ that the community finds out about fracking. The farmer is shocked and ‘begins to sway’, drawing a temporal boundary between before and after constituted by his emotional reaction. As well as indicating the arrival of fracking, it suggests the end of an Edenic state of Nature which the performance begins with. This natural idyll—largely visualized through romantic pastoral images of what campaigners fear will be lost—is disrupted and unsettled by fracking. Participants described how Leitrim involved ‘a different way of life’. ‘It’s peaceful and it’s quiet. And if this goes ahead it will be finished forever.’ The future with fracking is typically described and represented in intense emotional terms, such as ‘total disaster’ and ‘Armageddon’, indicating an affective resonance with the scale and power of the proposed technology.

My suggestion is that intense affective practices such as shock play an active technopolitical role in shaping and materializing SIs. Jasanoff (2015, p. 4) has observed how ‘scientific and technological visions enter into the assemblages of materiality, meaning, and morality that constitute robust forms of social life’. Affect is an important dimension in this respect. As Campbell et al. (2017, p. 610) argue, ‘without emotions it is difficult to frame and resolve meaningful moral questions … memory and imagination require an emotional commitment to develop and mark meaning, genuineness and relevance’. Shock and other intense emotions like fear and disgust contribute to the normative ordering of time, space, and society. An Edenic past and fracked future, a fractured landscape, and an innocent threatened community all take shape through this affectively infused sociotechnical imaginary. From Wetherell’s (2012) perspective, intense emotions are crucial in this process because they are ‘the material from which people select and build more global subjective feelings of interactional and relational direction and thrust’ (p. 84). This, then, helps us to understand one of the key things that SIs are supposed to help us understand: ‘why, at significant forks in the road, societies opt for particular directions of choice and change over others and why those choices gain stability or, at times, fail to do so’ (Jasanoff, 2015, p. 14).

Affect plays a role in the creative and productive function of imaginaries. In the case of fracking, shock and fear unsettle the community’s understanding of itself and its relationship to the time and space it inhabits, and were a driving force behind shared visions of the future. It was not all negative, however: Shock and fear spurred the community on to imagine alternative technosocial futures. As one campaigner shared with me:

For us as members of the community, to be stakeholders in an energy company where we are employing and getting a dividend … to get that mill wheel back up and running and build a sustainable living centre there where people can come and train … so, more of that happening and communities and villages taking control of their own lives.

Fear is tied in a complex relationship with hope. The hope, in one sense, is dependent on the fear. We might ask whether this vision of a sustainable future would have arisen if the threat of fracking had not arrived, indicating that the relationship between shared moral values and collective visions of the future can involve a layered and interactional series of affective practices. This is worth considering if we want to understand the productive dimension of the imaginary, especially when it comes to questions of power and agency. Powerful testimonies, images of harm, and acknowledgement of suffering help guide our decisions about where we need to act and how quickly. It is important to point out here that emotional intensity is not an inherently superior moral index of imagination. These affective practices are deeply political (Ahmed, 2014). Cherry (2021) for example, outlines the importance of rage in the anti-racist struggle. Paying attention to these emotions and experiences can help us to understand, in greater depth, what relationships of power are doing, what the stakes are in a given situation, whose bodies are on the line, and how we ought to respond. Without ascribing a universal truth to collective emotions, we can recognize that they are powerful, they do stuff, and that affective practices like shock and fear order and shape imaginaries.

### Multimodality

**Figure 1.**
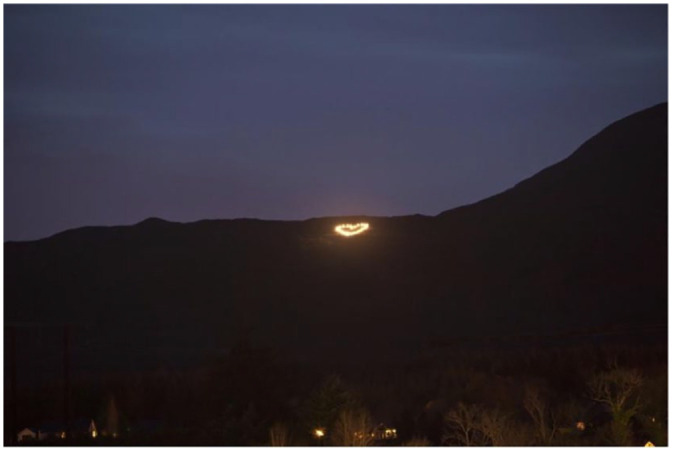
‘The Heart on the Hill’ art installation on Benbo Mountain above Manorhamilton, County Leitrim. Photo credit: Joseph Sheerin.

‘The Heart on the Hill’ was an art installation on Benbo, a small mountain outside Manorhamilton in north Leitrim (Figure 1). Designed by a local artist and assembled by *Love Leitrim*, the installation comprised of a number of bright LED lights strung across the side of the hill in the shape of a heart. The Heart on the Hill was reinstalled in subsequent years before Christmas and to celebrate the banning of fracking in Ireland. It was also assembled for the Stony Woods festival in Kiltyclougher, an event commemorating the lives of people in the area who passed away young. One campaigner explained that the idea was ‘to get it in a spot where as many people as possible could see it from the area of Manorhamilton’. They went on to say that ‘communities have their differences, but when they all agree on one thing it’s always a good thing too, so to try and bring people together in a community, it’s nice to have something everybody has in common that they like, as well as living here.’ A local newspaper, *The Leitrim Observer*, described the event as ‘a truly community effort’ (Heavey, 2016). We can understand the role of affect here as ‘onto-formative’, meaning that it ‘constitutes subjects and objects’ (Wetherell, 2012, p. 159). Crucially, however, affect is not to be understood as something that is separate from subjects and objects, but as a relational practice which contributes to their organization, and is important when considering the roles that ‘science and technology play in connecting the individual’s subjective self-understanding to a shared social or moral order’ (Jasanoff, 2015, p. 5).

We can see the organization of subjects and objects in how the Heart on the Hill installation was woven into daily practices like commuting. ‘There were people who said they were travelling to and from work every day, they’d come back from Sligo after a busy day of work and it was just nice that on their way home to see the Heart on the Hill nearby to where they live.’ Affective practices are moments of ‘recruitment and often synchronous assembling of multimodal resources, including, most crucially, body states’ (Wetherell, 2012, p. 159). *Love Leitrim* and love for Leitrim are gathered together as the Heart on the Hill is embedded in the community’s routine and everyday practices. Importantly, the Heart on the Hill is also attached to powerful individual and shared embodied experiences of mourning, loss, and hope.

One participant explained the Stony Woods Festival to me as ‘a remembrance festival particularly for young people who have died from around this area’. After the first installation of the Heart on the Hill, the organizer of the Stony Woods Festival asked *Love Leitrim* to install the heart as part of the festival.

Again, it went down very well—especially with the people who had lost family members—they kind of felt it was significant for them so, and the final part of that particular festival on the Sunday night was a lighting of sky lanterns, the candles, they sent up one of them for each person that had been lost and it was a nice way to end the festival. That they did that with heart on the hill in the background.

Other people, [a neighbour] for example here, who lost a son, she used to see it regularly travelling the road and she just said it used to lift her spirits, she’d always think of her son when she saw it on the hill so. Just very meaningful and moving things came back to us from different people.

More than a symbol of love for those living in Leitrim, the Heart on the Hill can be understood as a material and spatial practicing of love and grief where powerful individual and shared feelings are recruited and assembled by the lights, memories, and mountains. The land threatened by fracking is mobilized as a technopolitical site of resistance. Wetherell (2012) argues that meaning emerges through ‘the direction and history of affective practice over time, and the history of its entanglements with other onto-formative social practices and social formations’ (p. 159). The Heart on the Hill echoes the Irish cultural practices of Bealtainne and the Samhain Fire Festivals, where fires were lit on high to mark the contrast between brighter and darker times of the year (Butler, 2004). Butler makes the connection between the lighting of fires at Samhain today [Halloween bonfires] and the ancient pre-Christian pagan practices of lighting fires on hills of the Irish landscape. Samhain is ‘an affirmation of life and vibrancy in the face of the coming dark and harshness of winter’ (Butler, 2004, p. 69). This resonates with the Heart on the Hill in the affective context of fracking, in that it was erected during the winter before Christmas and enacted as a beacon of hope for the community. These beacons served as a confrontation of physical darkness and ‘personal metaphorical darkness, sometimes confronting challenging emotional issues’ (Butler, 2004, p. 69).

The mountain, solar-powered LEDs, the figure of the heart are all mobilized to generate material significance. Keeping the installation visible on the mountainside kept affects of love circulating and tied them to a physical place. Love is not just felt—it becomes a situated, spatial relationship, practiced through looking at the distant mountain overlooking Manorhamilton. It also gathers the community, marking out a special domain within which they share love for the area. In this way, it provides a closed space of intimacy in which the ‘self’ of the community is gathered. It is necessary to stress the relational back-and-forth nature of affective practice. The community is not simply taken up and drawn one way or another by affective practice. Emotions like love and their embeddedness in festivals and routine practices have a practical orientation. Affective practice involves ‘practical methods for performing, re-animating and re-configuring social relations’ (Wetherell, 2012, p. 115). The fact that these practical emotional methods are multimodal allows us to broaden the registers of meaning through which we understand and analyse imaginaries. From this perspective, collective imagination is not simply discursive: it involves the emotive meaning-making of human bodies and the material contexts that those bodies are situated within. Of course, the affective dimension of imaginaries will frequently be discursively mediated, where emotions are ‘completed in discourse’ (Burkitt, 1997, cited in Wetherell, 2012, p. 24). However, we should become more aware of the role that the body and subjectivities play in shared imagination and resistant orderings of technosocial value.

### Dynamics

In this section, I argue that love and hate are dynamic; they are the outcome of conflicts and tensions that are constantly managed and defended against. I suggest that this troubles a straightforward mapping of love or hate onto a single desired or feared future. In examining the dynamic interplay of love and hate in the *Love Leitrim* campaign, I argue that the conflicts that underpin these emotions need to be experienced (via emotional growth) if the group is to develop a sociotechnical imaginary that is capable of critically reflecting on its own values and purposes (the utopian function of imaginaries).

*Love Leitrim* worked to establish the dichotomy of love and hate through their campaign through signs, t-shirts, banners, and stickers with slogans such as ‘Love Leitrim/Hate Fracking’, ‘Love Music/Hate Fracking’, and ‘Love Football/Hate Fracking’. The name of the campaign itself emphasizes the group’s commitment to what is loved about the community, place, and way of life in opposition to the threat of fracking. In contrast, fracking and the groups associated with it were hated, to varying degrees. From this perspective love seems self-evidently aligned with Leitrim and hate is aligned with fracking. However, this representation of static and self-evident emotions does not match the day-to-day practices and talk about emotions, which revealed them to be far more conflicted and managed.

One conflict related to the danger that the fracking campaign posed to peace in communities North and South of the border. One campaigner articulated this conflict: ‘Everybody was terrified. Absolutely terrified that there would be a return to that [Troubles violence].’ Others shared this fear: ‘I remember the first presentation I ever gave about fracking … and a guy down the back of the hall just stood up and said, “just let us know when they’re here” and sat back down again … this will turn into a recruitment zone for paramilitaries if they try to impose a project here.’ Another campaigner told me, ‘[o]ur communities are threatened with fracking—we’re six kilometres from the border here. … [I]t has not been easy through The Troubles to be a border town, to be a border dweller, both economically and emotionally and spiritually.’ I was told that ‘there aren’t any bombs going off, but the two communities are as separate, really, as they ever were’. Political division created tensions and had a practical impact on the ability of communities to work together in opposition to fracking. A campaigner explained how ‘it inevitably became almost a sectarian issue—that fracking was something that the Unionists supported, and Nationalists opposed’. In this conflict, *Love Leitrim*, the organization representing what is loved (and moved towards) threatens to bring about violence and disunity (what is feared and resisted).

Campaigners also discussed a second conflict, whereby fracking—the thing that is hated (and resisted)—brought about unity (which is desired). ‘There was years and years of money ploughed into the border area through the peace programs—the threat of fracking did far more to bring us all together.’ Another campaigner outlined how fracking sewed together sectarian divisions: ‘Even in Fermanagh district council, that it did vote no … and that was cross-community … it was accepted that it is a cross-community campaign.’

My suggestion is that these conflicts (loving and hating the same object) are painful and require the employment of management strategies to defend against that discomfort. One type of defence is splitting and idealization. This involves campaigners positioning *Love Leitrim* as an all-positive group that is defined by love and a near-total absence of hate. Conversely, certain groups (e.g., government, EPA, oil and gas industry) are positioned as being entirely callous, corrupt, or hateful. Alford (1989) argues that

the best indicator of a group’s [split] character is not its hatred and aggression (which are often suppressed) but the degree of idealization it practices. Excessive idealization—of one’s own group, values, or favorite person (for example, God)—is almost always a sign that intense persecutory fears are bubbling under the surface. (p. 88)

We can see community attempts to split off the campaign from its violent potential in the following participant’s remark: ‘We didn’t want to be anti-anything—we are anti-fracking, but we didn’t want our name to be associated with something negative.’ Another explained that *Love Leitrim* was about ‘pushing the positive’. The dance performance described earlier visualizes the split between who is to be loved and hated. In representing the community as farmers and animals, the campaign is aligned with values of purity and goodness and associated with nature and tradition. In representing government and industry as people in suits carrying money signs, the opposition is aligned with corruption and greed and associated with colonial modernity (Gibbons, 1996). The identity of the hated other was also clearly represented in messages that visitors to a sculpture exhibition had left on one of the venue’s walls. These writings and drawings, which visitors had been invited to create, included phrases such as: ‘frack off frackers’, ‘frack the frackers’, ‘we hate fracking’, and ‘for fracks sake’. It is important to note that splitting does not mean that the community was not actually being persecuted by greedy or corrupt figures or that the locals were not really worthy of love. Rather, the unconscious management of emotions can serve to disproportionately present relationships in a static, universal, and polarized way that misses their dynamic tensions and conflicts.

Moreover, this unconscious emotional work arguably serves to displace the environmental-technological issue at the heart of the debate. Anxieties about fracking are displaced and attached to a range of groups outside of the community, shifting the focus of debate to a conflict between the self and other. This is complicated by the dynamics of projection, whereby the neat division between the ‘goodness’ of the community and the ‘badness’ of those outside of it is undermined by an unconscious acknowledgement of the harm that the campaign itself might cause in opposing fracking. The anxiety caused by this acknowledgement only serves to drive the wedge between self and other further, as any anger towards the campaign group from members within that group ‘is split off as aggression against other groups in order to allow a more secure, dependent attachment to one’s own group’ (Alford, 1989, p. 67).

By promoting love and positivity in an attempt to disavow the potential violence that the campaign might bring, the campaign was in danger of producing the kind of utopic imaginary that Thaler (2022) cautions against: an imaginary that succumbs to magical thinking or totalitarianism, by simply erasing the conflicts and tensions or replacing them with a singular, idealized technosocial arrangement (i.e., that of *Love Leitrim*). Imagining the community in all-good terms offers only a partial view of the campaign by focusing on its positive aspects at the expense of parts that might be in conflict. The reality of energy consumption is an uncomfortable one. Groups need to confront the fact that energy resources are limited, that producing energy is resource intensive and requires a lot of labour, that energy flows are unpredictable, and that there are typically diverging interests and perspectives on which energy sources are best. Learning how to live with and manage diverse energy needs in a rural community—a border community, no less—requires the acknowledgement and processing of conflicting emotions. A binary theory of desire/fear or love/hate ignores this conflict. Acknowledging conflict allows groups to appreciate their own destructive capacities, apprehend others on their own terms, recognize interdependence, appreciate environmental limitations, and acknowledge diverse needs and perspectives (both from within the group and outside it). Without an acknowledgement of these emotional dynamics, the desired sociotechnical future—the ‘energy company where we are employing and getting a dividend’, the ‘mill wheel’, and the ‘sustainable living centre’—risks falling into wishful fantasies or totalitarianism, whereby conflict is magically erased in favour of a single valorized perspective. This can usher in the kind of magical thinking that sees the community through a romantic prism, rather than in terms of its actual needs. It also risks lacking perspective on the diversity of needs that the imagined future should incorporate. In focusing only on those who contribute positive or loving emotional energy to the campaign, others who are dissatisfied or not served by the campaign are excluded.

Confronting the difficult emotional aspects of an imaginary requires an integrated emotional perspective, a perspective capable of ‘enabling loved and hated attributes to be recognized as belonging to the same object’ (Rustin, 2013, p. 177). Here, the conflicts are experienced, tolerated, and worked through, rather than erased through defensive techniques like splitting. This is a kind of ‘emotional learning’ that can ‘lead to the release of repressed emotions’ (Alford, 1989, p. 69). When these emotions are released or ‘made public’, they can then be integrated by the group ‘in a way that repressed and split-off emotions never can be’ (Alford, 1989, p. 69). Defences, when successful, protect us from experiencing the very conflicts that we must face if we are to imagine and build a practical and just technosocial world. Emotional learning is the process through which a group develops the capacity to tolerate ambivalence and conflict rather than seeking to immediately overcome it. For Thaler (2022), the utopian function of imaginaries—their capacity to make things otherwise—requires an ‘education of desire’. This education involves ‘not only the production of scientific knowledge, but also the cultivation of imaginative proposals that allow us to reassess, emotionally and bodily, how we should live in a “world of shared inhabitance”’ (Thaler, 2022, p. 23). Emotional learning is essential to achieve the kind of ‘critical self-reflexivity’ that Thaler argues is ‘pivotal to the orientating function that all utopias perform’ (p. 50). An important aspect of this emotional growth is a drive towards repair.

Alford (1989) argues that defences like splitting discourage ‘the integration of good and bad necessary for the development of reparative psychology and morality’ (p. 89). Emotional growth puts us in a position that ‘confronts, rather than transcends, the sheer ugliness of so much in the world’ (Alford, 1989, pp. 107–108). In the context of *Love Leitrim*, this ugliness includes the group’s destructive capacity to reignite the violence of The Troubles, the profit-motives of energy companies, the Leitrim community’s perceived dependence on the government, the environment, and energy (from whichever source), and diverging opinions within the campaign group itself. From the psychoanalytic psychosocial perspective, it is only by confronting these conflicts that the group can begin the process of repairing them. There is an unavoidably practical—realistic—aspect of the reparative, utopian sociotechnical imaginary, in that it needs to be ‘roughly in accord with the objective demands placed on the group, its task in the world’ (Alford, 1989, p. 102). That task is working out how to respond to the prospect of fracking by proposing an alternative energy imaginary. This comes back to Thaler’s (2022) argument for the need for an education of desire:

If social and political factors have a bearing on how we move around the world, how we stand in relation to different objects, how we become (or fail to become) affected by those near to us, then we should reflect on the ways in which these factors can be transformed in light of specific objectives. (p. 23)

*Comhrá* (Irish for ‘conversation’) was an initiative set up to address the marginalizing effect of *Love Leitrim*. It aimed to repair relationships within the campaign. As a campaigner explained, ‘big monster meetings in the community’ were felt by some to be hierarchical and intimidating, with ‘a lot of language people weren’t sure of’. Inspired by the ‘Lock the Gate’ movement in Australia, the *Comhrá* subgroup set about meeting with the community on a door-to-door basis, asking them what they want for the future of their county. Rather than assuming what everyone wanted, *Comhrá* sought to let the people lead. I was told that *Love Leitrim* didn’t want to assume what other people wanted for the area. One campaigner made clear that their vision might not be shared by others: ‘It could be a completely different thing than I’m thinking, it could be that, you know, it could be anything, all the older generation might be saying “ah no sure we have to let this place go to hell, we should all move to Manorhamilton, we can’t be living like this anymore, that’s mad.”’ Here, we see *Love Leitrim* consciously acknowledging the destructive homogenizing capacity of the group and engaging in emotional learning to change in response to potential internal conflicts. The conflicts don’t go away, but the group can avoid the totalitarianism and magical thinking of a single future by acknowledging how dynamic emotions are managed and defended against. ‘[T]hinking with and through our discomforts’ (Shanley et al., 2022, p. 125) can open up space to reflect on issues of care and responsibility in science and technology.

The affective technopolitics of SIs exists in large part in its utopic function, in that the ‘dreamscapes of modernity’ circulate through ‘grand aspirations and adventures with science and technology’ (Jasanoff, 2015). These dreamscapes are affectively polarized ‘between positive and negative imaginings—between utopia and dystopia’. However, as we have seen, this binary setup neglects the conflicted and ambivalent way that affect is typically experienced and performed and the manner in which shared desires and fears are negotiated. Illuminating how emotional conflicts are managed and defended against, and how they are projected as all-good or all-bad technosocial arrangements, positions us to better understand and evaluate different sociotechnical imaginaries.

## Conclusion

Emotions are typically presented as self-evident black boxes that lack a clear account of their capacity to power change. Affect is isolated from everyday practices of meaning-making, obscuring how their multimodality draws collectives together around a specific technoscientific concern. Emotions are usually presented in binaries, restricting their function to drawing individuals and groups towards or away from a given future. In this article, I’ve attempted to demonstrate how attention to the intensity, multimodality, and dynamics of emotions provides access to a more nuanced account of the technopolitics of shared imagination.

The scale and power of a technology like fracking produces powerful emotional responses, such as shock and disgust, and how these affective practices order space and time in the creation of alternative sociotechnical futures. The landscape that was threatened by fracking was repurposed with the Heart on the Hill installation, drawing a collective together in opposition to fracking through multimodal affective practices of love and grief. And seemingly self-evident emotions like desire and fear obscured a more complex set of unconscious conflicts whose management required careful attention to achieve a more reparative and just utopian vision.

This has broader implications for STS, particularly for our understanding of affect in relation to questions of humanity, representation, and building more just technosocial worlds (de la Bellacasa, 2011; Groves et al., 2016; Wilson 2015). For one, attention to affect illuminates how the intensity of emotional experience produces sociotechnical change through distinctive forms of human creativity and imagination. This offers insights on the distinctiveness and diversity of being human in a technosocial world. For Jasanoff (2010, p. 234), it is the fine-grained ‘thick description’ of our ‘emotional and spiritual existence’ that gives meaning to our lives as ‘fully-formed human being[s]’. The biological human body plays an important role in the politics of imagination, producing the possibilities for creativity that allow us to imagine otherwise (McNeil et al., 2017). However, our bodies are fragile and vulnerable and frequently let us down, reminding us of our limitations, our mortality, and our dependence on others and our material environment. Fragility, suffering, dependence, and death are essential, if difficult, aspects of human existence. Against fantasies of transcendence, immortality, and limitless freedom and choice, these insights into our essential humanity call on us to recognize the inherent differences and limitations that the social world produces on our bodies and to use this insight as an opportunity to imagine more caring and inclusive worlds. Work on ‘disability imaginaries’ indicates how this can be done, arguing that how we imagine the future must be ‘grounded in an understanding of human persons and communities as embodied, diverse, and constituted by their social, historical, and material circumstances’ (Karpin & Mykitiuk, 2021, p. 13).

We might also think of how the multimodal and embodied nature of affective imagining opens sociotechnical analyses up to a broader palette of representations and meanings. A wider range of meanings and valences can help us more accurately capture how different collectives negotiate and articulate technoscientific forms of life. Beyond the typical discursive modalities with which sociotechnical imaginaries are examined (e.g., reports, legislation, written historical archives) analysis of collective visions can be traced through gatherings (e.g., protests, cultural events), communal rituals and practices, artworks, movies, or websites. This can yield richer accounts of meaning and identify broader kinds of representation, both in terms of how knowledge is articulated and made sense of and how communities are recognized and included in discussions and decision making (Chilvers & Kearnes, 2015). This is essential if STS truly aspires to be inclusive of non-Western and indigenous forms of knowledge and practice in the making and remaking of new forms of life (Todd, 2016). As an example, we might look to how researchers in New Zealand have used an indigenous Wairau approach to do research (Moewaka Barnes et al., 2017).^4^ Moewaka Barnes et al. (2017) demonstrate how affective practice offers ‘resonating pathways and ideas’ (p. 316) for exploring Wairau as a unique and distinctive approach to research. Wider meanings allow us to capture how different collectives represent the future and are represented in it.

Finally, attention to the dynamics of the technopolitical unconscious reveals how collectives imagine sociotechnical worlds within a context of conflict and ambivalence, and how these conflicts are managed and worked through shapes how just those worlds will be. Working through the emotional dimension of shared visions can allow collectives to critically reflect at a deeper level and access more complex and nuanced forms of social engagement like reparation and care. Achieving reparative and caring imaginaries takes emotional learning and growth. Indeed, as Groves et al. (2016) argue, the emotional effort required to overcome discomfort is the very definition of ‘care-full engagement’ (p. 11).

## Notes

1. Fracking is the popular term for hydraulic fracturing, a technique used for gas exploration and extraction. It is used to mine for unconventional gas, i.e., gas that comes from unconventional sources such as ‘shale gas deposits, coal seams, and tight sandstone’ below the surface of the ground (Hooper et al., 2016, p. ix). A bore hole is drilled and filled with fluids—generally water, sand, and other chemicals (Chen & Gunster, 2016, p. 310)—until enough pressure builds to cause the rock to fracture and allow trapped natural gas to flow up to the surface well head for capture (Healy, 2012, p. 4).

2. There are numerous routes into an STS-friendly theory of affect. Work within STS that explicitly deals with affect, such as de la Bellacasa’s (2011) ‘matters of care’, Haraway’s (2004) theory of posthuman embodiment, or Wilson’s (2015) gut feminism provide useful conceptions of emotion and the body. Other work from the humanities and social sciences has also been useful for STS scholars, such as affect theory (Clough, 2008), non-representational theory (Thrift, 2008), or affective economies (Ahmed, 2014). I have opted to focus on affective practice and psychoanalytic psychosocial studies, as I believe that these two approaches harmonize with many of the above perspectives while also working particularly well with the material-practical aspect of imaginaries (affective practice) and the fantasy element of imaginaries (psychoanalysis).

3. This, of course, is not all that is required to address climate change. Climate change is a technopolitical issue, involving institutions, infrastructures, collectives, and materialities. Much needs to be done at these levels to facilitate meaningful change. However, when we consider the level of the imaginary—and the psychosocial *meanings* that are involved in collective imagination (Jasanoff, 2010)—we need to recognize that human subjectivities, emotions, discourses, and representations are present in the board rooms of fossil fuel companies, climatology labs, think tank meeting rooms, and government offices.

4. Wairua can be understood as ‘a Māori (indigenous peoples of Aotearoa New Zealand) concept, somewhat restrictively translated as spirit or spirituality’ (Moewaka Barnes et al., 2017, p. 313).
